# By-Products from Winemaking and Olive Mill Value Chains for the Enrichment of Refined Olive Oil: Technological Challenges and Nutraceutical Features

**DOI:** 10.3390/foods9101390

**Published:** 2020-10-01

**Authors:** Monica Macaluso, Alessandro Bianchi, Chiara Sanmartin, Isabella Taglieri, Francesca Venturi, Lara Testai, Lorenzo Flori, Vincenzo Calderone, Marinella De Leo, Alessandra Braca, Valerio Ciccone, Sandra Donnini, Luca Guidi, Angela Zinnai

**Affiliations:** 1 Department of Agriculture, Food and Environment (DAFE), University of Pisa, Via del Borghetto 80, 56124 Pisa, Italy; monica.macaluso@phd.unipi.it (M.M.); dr.a.bianchi91@gmail.com (A.B.); chiara.sanmartin@unipi.it (C.S.); angela.zinnai@unipi.it (A.Z.); 2 Interdepartmental Research Center “Nutraceuticals and Food for Health”, University of Pisa, Via del Borghetto 80, 56124 Pisa, Italy; lara.testai@unipi.it (L.T.); vincenzo.calderone@unipi.it (V.C.); marinella.deleo@unipi.it (M.D.L.); alessandra.braca@unipi.it (A.B.); 3 CISUP, Centre for Instrumentation Sharing Pisa University, Lungarno Pacinotti 43, 56126 Pisa, Italy; 4 Department of Pharmacy, University of Pisa, Via Bonanno Pisano 6, 56126 Pisa, Italy; ciccone3@student.unisi.it; 5 Department of Life Sciences, University of Siena, Via Aldo Moro 2, 53100 Siena, Italy; lorenzo.flori@phd.unipi.it (L.F.); sandra.donnini@unisi.it (S.D.); 6 Toscana Life Sciences Str. del Petriccio e Belriguardo 35, 53100 Siena, Italy; 7 SALOV S.p.A., Via di Montramito, 1600, 55054 Massarosa, Italy; luca.guidi@salov.com

**Keywords:** phenol-enriched olive oil, grape marc, olive pomace, olive leaves, phenols, in vitro model, in vivo model, cardiovascular diseases, cancer diseases, metabolic syndrome

## Abstract

A growing body of literature is available about the valorization of food by-products to produce functional foods that combine the basic nutritional impact with the improvement of the health status of consumers. In this context, this study had two main objectives: (i) An innovative multistep extraction process for the production of a refined olive oil enriched with phenolic compounds (PE-ROO) extracted from olive pomace, olive leaves, or grape marc was presented and discussed. (ii) The most promising PE-ROOs were selected and utilized in in vitro and in vivo trials in order to determine their effectiveness in the management of high fat diet-induced-metabolic syndrome and oxidative stress in rats. The best results were obtained when olive leaves were used as source of phenols, regardless of the chemical composition of the solvent utilized for the extraction. Furthermore, while ethanol/hexane mixture was confirmed as a good solvent for the extraction of phenols compounds soluble in oil, the mix ROO/ethanol also showed a good extracting power from olive leaves. Besides, the ROO enriched with phenols extracted from olive leaves revealed an interesting beneficial effect to counteract high fat diet-induced-metabolic disorder and oxidative stress in rats, closely followed by ROO enriched by utilizing grape marc.

## 1. Introduction

In last few decades the increasing popularity of the Mediterranean diet (MedDiet) can be explained by both its organoleptic properties together with the health benefits it confers [1]. In particular, the high intake of vegetables, fruits, legumes, nuts, cereals, and monounsaturated fat (e.g., olive oil) that characterize this dietary pattern has been associated with positive effects in improving cardiovascular health together with a reduction of both the cognitive decline and the risk of development of Alzheimer’s disease [2]. The beneficial effects were shown by the MedDiet also in the “Metabolic Syndrome” (MetS) and are mainly due to the antioxidant and anti-inflammatory properties of the phytochemical components including phenols, mono- and poly-unsaturated fatty acids (MUFA and PUFA), tocopherols [3,4].

In the frame of MedDiet, the main source of fat is represented by virgin olive oil (VOO) that can be defined as a functional food endowed with a healthy profile thanks to the pleiotropic effects showed by tocopherols and MUFA (represented by oleic acid) together with the phenolic fraction [1,5].

According to the European Union legislation [6], olive oil is classified into four categories reflecting its quality and sensorial properties. Among them, ROO (refined olive oil) is a low-quality oil that undergoes chemical or physical refining to become edible and it is gaining importance in the food industry [1,7,8]. Fatty acid composition is similar in all the commercially available types of oils but minor components, mainly phenolic compounds that are assumed to be one of the healthiest fractions of olive oil, are depleted in ROO as they are lost during the extraction and refining procedures [1]. Due to the reduced or totally absent content of phenols [9,10], ROO is unstable and subjected to rapid oxidation during storage [8,11,12,13]. Furthermore, since organoleptic properties of ROO are very poor, it is usually commercialized and consumed as a component of mixed olive oil, which is ROO with a low percentage (10–15%) of VOO to improve palatability.

Given their variable composition, a large variety of mixed olive oil (OO) can be found in the market, with not only quite different total polyphenol content but also quite different polyphenol compositions [14]. Thus, a good strategy to ensure an optimal intake of polyphenols through habitual diet would be to produce enriched olive oil with well-known bioactive polyphenols [15,16,17,18,19,20,21].

On the other hand, the oxidative degradation of lipids, with particular regards to the oils with minimal or no phenol content, represents a main concern for the shelf life assessment of a great part of foodstuffs [3,13,14] so, in recent decades, the addition of external antioxidants in the recipes to slow down as much as possible the lipid degradation in food material, has gained growing attention [22,23]. In this context, while synthetic antioxidants (i.e., butylhydroxyanisole, butylhydroxytoluene, *tert*-butylhydroquinone) have been used as food additives to overcome the stability problems of oils and fats, recent reports reveal that these compounds may be implicated in many health risks, including carcinogenesis [24]. Due to these safety concerns, there is an increasing trend among food scientists to substitute these synthetic antioxidants with natural biological active substances mainly extracted from plants and vegetables as well as food by-products (i.e., raw materials derived from the same olive tree or the olive mill by-products) [25,26].

In the last few years, a growing body of literature has become available about the valorization of olive pomace, olive leaves, and grape marc as a source of valuable antioxidant compounds to be used to produce functional foods in a circular economy concept [25,26,27,28,29,30]. Besides, several studies have focused on the optimization of new methods for the production of OO and ROO enriched with natural antioxidants (namely phenols, tocopherols, carotenoids) extracted from different food wastes [12,13,26,31]. However, to the best of our knowledge, the main issues related to the industrial production of phenol-enriched refined olive oil (PE-ROO) are yet to be solved and no data is available in the literature on the feasibility of the use of grape marc, olive leaves, and pomace as a source of safe natural antioxidants to be used with this specific aim.

According to Sanchez-Medina and coworkers [18], there are three alternatives in the literature for oil enrichment with these high added value compounds from food by-products: (1) liquid–liquid extraction, in which the oil is put into contact with an alcoholic extract of phenols, which are transferred to the oily phase as a function of their distribution factor, removing the alcoholic phase by centrifugation; (2) solid–liquid extraction, in which the purified phenolic extract is dried under appropriate conditions and the paste obtained is partially dissolved into the oil as a function of the solubility of the different paste components in the oily phase; (3) a combination of these procedures, in which the alcoholic extract and the oil are put into contact and the two-phase system is subject to alcohol removal in a rotary evaporator.

In this context, this study had two main objectives:

(i) In the first part of the paper an innovative multistep extraction process for the production of a PE-ROO based on the use of the ROO as a component of the extraction mixture to recover high-added-value phenolic compounds from olive pomace, olive leaves, and grape marc was presented and discussed.
(ii) To verify if the nutraceutical value of the enriched olive oils was really improved, in the second part of the research the most promising PE-ROOs were selected and utilized in in vitro and in vivo trials in order to determine their effectiveness in the management of high fat diet-induced-MetS and oxidative stress in rats.

## 2. Materials and Methods

### 2.1. Chemicals

Acetic acid, ethanol, sodium carbonate, ethoxyethane, iso-octane, chlorane 37.0%, sodium hydroxide 0.1 N, sodium thiosulphate 0.01 N, potassium iodide, starch indicator solution 1.0%, ABTS (2,20-azinobis(3-ethylbenzothiazoline-6-sulphonic acid)), 4-(2-Hydroxyethyl)phenol, Trolox (6-hydroxy-2,5,7,8-tetramethylchroman-2-carboxylic acid), TrisHCl (2-amino-2-(hydroxymethyl)propane-1,3-diol chlorane), and lithium perchlorate (LiClO_4_) were supplied by Sigma Aldrich (Milan, Italy). 3,4,5-Trihydroxybenzoic acid was purchased from Carlo Erba (Milan, Italy). 3,3-bis(4-hydroxyphenyl)-2-benzofuran-1(3H)-one 1% and Folin–Ciocalteau reagent were obtained from Titolchimica (Pontecchio Polesine, Italy). Methanol, hexane, and formic acid for HPLC analyses were purchased from VWR (Milan, Italy). HPLC grade water (18 mΩ) was obtained by a Mill-Ω purification system (Millipore Corp., Bedford, MA, USA). CelLytic™ MT Cell Lysis Reagent was obtained from Life Technologies (Carlsbad, CA, USA). Streptavidin-conjugated HRP,3,3-diaminobenzidine tetrahydrocloride (DAB), Eukitt^®^ quick-hardening mounting medium fibrinogen, thrombin and VEGF were from Merck KGaA (Darmstadt, Germany). Tissue-Tek O.C.T. was from Sakura (San Marcos, CA, USA). Anti-COX-2 was from Origene (Rockville, MD, USA) [TA313292]; anti-ALDH1A1 and anti-4-HNE were from Abcam (Cambridge, UK) [ab9883 and ab46544]; anti-Catalase and anti-β-actin were from Merck KGaA (Darmstadt, Germany) [C0979 and A2228]; anti-mPGES-1 was from Cayman Chemical (Ann Arbor, MI, USA) [160140]; anti-iNOS, anti-eNOS, p65, p22-phox, and anti-CD40 were from Santa Cruz Biotechnology (Dallas, TX, USA) [sc-7271, sc-8008, sc-271968, and sc-975]. All reagents were analytical grade and were used as received without further purification.

### 2.2. Raw Material

The ROO utilized as a matrix to carry out the phenolic enrichment was produced by a physical refining process [13] at the industrial plant for vegetal oil refining SALOV S.p.A. (Massarosa, Italy).

Pomace and olive leaves (organic Moraiolo cv; organic Leccino cv) were collected at the end of a traditional olive oil extraction process described in a previous paper [32] performed by Spremioliva mod. C30, Mori-TEM srl, Italy.

Grape marc (organic Sangiovese cv) was collected at the end of a traditional wine making process for red wine production according to working conditions previously described [33].

To avoid microbiological spoilage and prevent the oxidative degradation of phenolic compounds, both pomace and grape marc were frozen and stored in inert atmosphere (N_2_ 100%) until use.

Total phenols of pomace, grape marc, and olive leaves are reported in Table 1.

### 2.3. Preparation of Phenolic Extracts from Pomace (P), Olive Leaves (OL), and Grape Marc (GM) and Phenol-Enriched ROO

**Figure 1:**
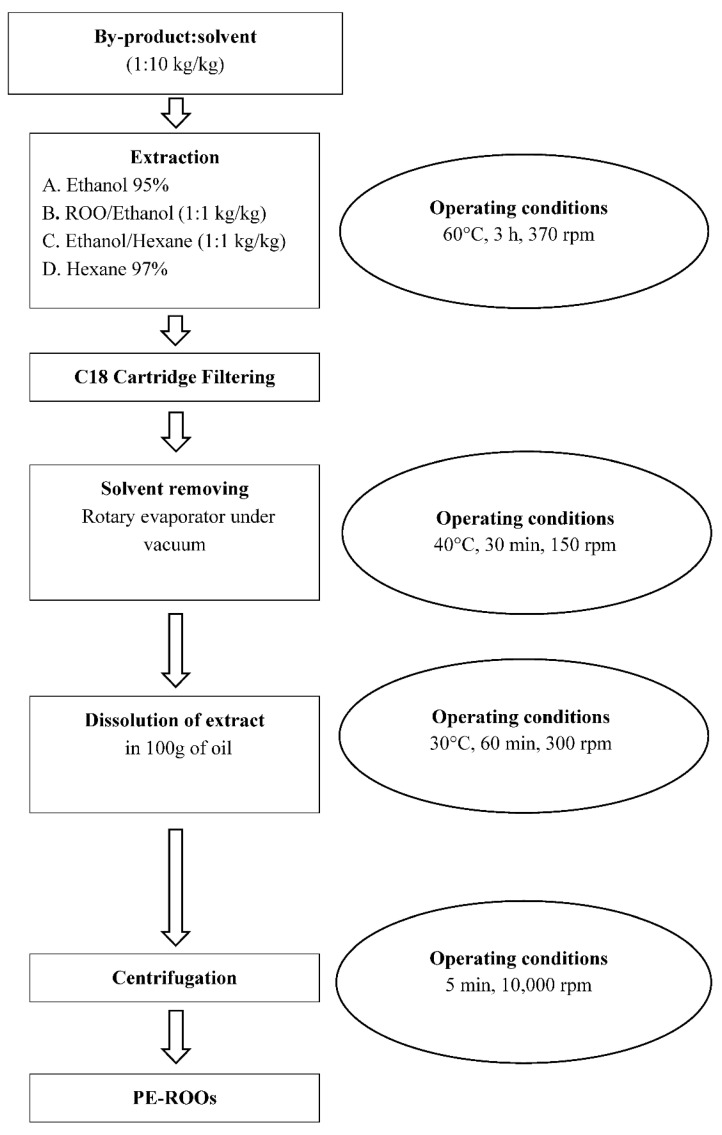
Experimental setup in which all extraction phases (extraction, filtering, solvent removing, dissolution, centrifugation, final product) and operating conditions (time, temperature, and extraction speed) of the recovery of antioxidant compounds from different by-products (olive pomace, olive leaves, and grape marc) are shown.

Based on the recent literature [12] and on our previous experience in the recovery of antioxidant compounds from different food by-products [13,23,34], the preparation of the PE-ROO was carried out according to the scheme reported in Figure 1 and further discussed.

To setup the recovery of the phenolic compounds from pomace, olive leaves, and grape marc, we studied different extraction processes by utilizing four different solvent solutions: Ethanol (95.0%), named sol. A; Ethanol/Refined Olive Oil (1:1 kg/kg), named sol. B; Ethanol/Hexane (1:1 kg/kg), named sol. C (Figure 1); Hexane (97.0%) named sol. D.

At the end of each extraction run, after a cartridge filtering (Sep-Pak classic C18, Waters^®^), the extract was rotary evaporated (Laborota 4000, Heidolph Instruments GMBH and Co. KG, Schwabach, Germany) until all solvents were eliminated; the resulting dry extract was then dissolved in 100 g of ROO (Figure 1).

Thus, 12 different phenol-enriched oils prototypes (PE-P-A ÷ PE-P-D, PE-GM-A ÷ PE-GM-D, and PE-OL-A ÷ PE-OL-D) were prepared (Table 2).

Finally, the phenol-enriched oil prototypes (PE-ROOs) were collected after centrifugation (IEC CL31R Multispeed, Thermo Scientific, Melegnano, Milan, Italy) at 10,000 rpm (16,770 g), 5 min, 15 °C, to eliminate any unsolved residues and maintained under inert atmosphere (N_2_ 100%) at 12 ± 1 °C until analysis.

### 2.4. Quality Parameters

#### 2.4.1. Free Fatty Acids, Peroxide Value, and Spectrophotometric Indexes

The general chemical parameters free fatty acids (FFA), peroxide value (PV), and spectrophotometric indexes (K_270_ and K_232_) of the starting ROO and PE-ROO were determined by acid base titration, iodometric titration, and spectrophotometric analysis in ultraviolet region (λ_270_ and λ_232_), respectively, according to the analytical methods described in the Regulation 1348/2013 of the European Union Commission and later modifications [35]. The analyses were performed at the laboratory of Food Technology of DAFE (University of Pisa, Italy).

#### 2.4.2. Phenolic Content

Total phenols were extracted from the oil as previously described [36]; the extracts were stored under inert atmosphere (N2 100%) at −20 °C until use. Total phenols concentration was measured by Folin-Ciocalteau colorimetric assay slightly modified: briefly, the extract (1 mL), Folin−Ciocalteu reagent (1 mL), and 7.5% sodium carbonate (9 mL) and deionized water (14 mL), were added to a 25 mL glass flask, mixed, and, after 120 min of incubation at room temperature, absorbance of the samples was measured at 765 nm against a blank. Total phenols content was expressed using a calibration curve prepared with gallic acid as the standard.

#### 2.4.3. Free-Radical Scavenging Capacity (FRSC)

FRSC was determined using both the DPPH assay (FRSC_DPPH_) [37] and the ABTS assay (FRSC_ABTS_) [38]. The Trolox dose–response curve used was in the (0.2–1.5) mM range. FRSC was calculated as Trolox Equivalent Antioxidant Capacity (TEAC) per mL of extract [23].

#### 2.4.4. Intensity of Bitterness (IB) Determination

Bitter components were extracted from 1.00 ± 0.01 g of oil samples using 6 mL extraction columns (Sep-Pak C18 Classic Cartridge, Waters s.p.a., Sesto San Giovanni (MI), Italy) and the IB was determined following the method previously described [39] recording absorbance at 225 nm.

#### 2.4.5. UHPLC-HR-ESI-MS/MS Analyses

The polar fractions of ROO control, PE-P-B, PE-GM-B, and PE-OL-B were obtained by extraction of each sample with MeOH-H_2_O (volume fraction 70%) using the procedure described in Flori et al. [37]. The obtained extracts were dried by rotavapor, dissolved in MeOH, and centrifugated (4000 rpm). Then, 5 μL of each extract was injected into an LC system composed by a Vanquish Flex Binary ultra-high-performance liquid chromatography (UHPLC) and a high resolution-mass spectrometer (HR-MS) Q Exactive Plus MS, Orbitrap-based FT-MS system, equipped by an electrospray (ESI) source (Thermo Fischer Scientific Inc., Bremem, Germany). UHPLC were performed on a Kinetex^®^ Biphenyl column (100 × 2.1 mm, 2.6 μm) provided of a Security Guard TM Ultra Cartridge (Phenomenex, Bologna, Italy). The elution was done by using a mixture of formic acid in MeOH 1 mL/L (solvent A) and formic acid in H_2_O 1 mL/L (solvent B) at a flow rate 0.5 mL/min. The solvent gradient was as follows: 0–10 min, 5–40% A; 10–21 min, 40–65% A. Spectra were acquired both in full (70,000 resolution, 220 ms maximum injection time) and data dependent-MS/MS scan (17,500 resolution, 60 ms maximum injection time), using ESI interface in negative ion mode (scan range of *m*/*z* 150–1200). The following ionization parameters were used: spray voltage 3400 V, capillary temperature 300 °C, sheath gas (N_2_) 35 arbitrary unit, auxiliary gas (N_2_) 8 arbitrary unit, collisionally activated dissociation (HCD) 18 eV. Xcalibur software was used for data processing.

### 2.5. In Vivo Evaluation of Nutraceutical Value of Olive Oils

In vivo experiments were carried out according to both European (EEC Directive 2010/63) and Italian (D.L. 4 March 2014 n.26) legislation (number protocol 144/2019-PR, 18 February 2019), as previously reported [37]. Briefly, adult male Wistar rats (3–4 months old, ENVIGO) with a body weight between 345 and 350 g were randomly divided into seven groups (five animals per group) and treated for 21 days. The first group was treated with a standard diet (STD, ENVIGO); the others were fed with a high fat diet (HFD, SAFE). In particular, the second group did not receive supplementations (HFD), the third group was treated with extra-virgin olive oil (HFD + EVOO) 5.5% kg/kg (HFD + EVOO, as reference group) mixed in powder feed [36,40]. The fourth group was treated with ROO 2% kg/kg mixed in powder feed. Then, the other groups were supplemented with novel olive oil formulations: PE-OL-B 2% kg/kg (in order to ensure the same intake of polyphenols as the EVOO, HFD + PE-OL-B), PE-P-B 2% kg/kg (HFD + PE-P-B), and PE-GM-B 2% kg/kg (HFD + PE-GM-B). After 3 weeks, at the end of the treatment, each animal was deprived of food and after 24 h was anesthetized with Thiopental Sodium (70 mg/kg, MSD animal health). The blood was collected, through the caudal vein, to perform a blood glucose test (Glucocard G meter, Menarini Diagnostics). Subsequently the animals were sacrificed with an overdose of Thiopental Sodium. Organs (heart, aorta, and abdominal adipose tissue) were removed, weighed, and stored for functional, enzymatic, and biochemical investigations.

#### 2.5.1. Functional Analysis of Cardiac Mitochondrial Membrane Potential

Hearts deriving from animals 21 days-treated were cut into small 2–3 mm^3^ pieces in ice cold isolation buffer (composition: Sucrose 250 mM, Tris 5 mM, EGTA 1 mM; pH 7.4) and processed as reported in Flori et al. [36]. Briefly, hearts were homogenized by Ultra-Turrax (model: IKA, T-18 Basic) and cardiac mitochondria were isolated through differential centrifugations. The pellet obtained, corresponding to the mitochondrial fraction, was resuspended in minimal volume of buffer and the mitochondrial protein concentration was determined spectrophotometrically by Bradford assay (Bio-Rad, Hercules, CA, USA), using a microplate reader (EnSpire, PerkinElmer, Waltham, MA, USA).

The membrane potential (∆Ψm) of the isolated mitochondria was determined using a potentiometric method. The lipophilic cation tetraphenylphosphonium (TPP^+^), used for this procedure, was detected with a TPP^+^ sensitive mini electrode (WPI, TipTPP, Sarasota, FL, USA), coupled to a reference electrode (WPI, Sarasota, FL, USA) and using data acquisition software (BiopacInc, Goleta, CA, USA). Mitochondria (1 mg protein/mL) were then added to the incubation buffer (composition: KCl 120 mM, K_2_HPO_4_ 5 mM, Hepes 10 mM, Succinic Acid 10 mM, MgCl_2_ 2 mM, TPP^+^Cl^−^ 10 μM; pH 7.4), and continuously stirred with a small magnet to generate a mitochondrial suspension.

The membrane potential value was calculated according to the following experimental equation derived from Nernst Equation (1):

(1) $$\Delta \Psi_{m}=60\;x\;\log\frac{\frac{V_{0}{\left[TPP^{+}\right]}_{0}}{{\left[TPP^{+}\right]}_{t}}-V_{t}-K_{0}P}{V_{m}P+K_{i}P}$$

where ΔΨ*m* is the mitochondrial membrane potential (mV), *V*_0_ is the volume of the incubation medium before the addition of mitochondria, *V*_t_ is the volume of the incubation medium after the addition of the mitochondria, *V*_m_ is the volume of the mitochondrial matrix (taken as 1 µL/mg protein), [*TPP*^+^]_0_ and [*TPP*^+^]*_t_* represent, respectively, the *TPP*^+^ concentrations recorded before addition and at time t, *P* is the protein concentration expressed in mg, and *K*_0_ and *K*_i_ are apparent external and internal partition coefficients of *TPP*^+^ (14.3 and 7.9 μL/mg, respectively).

#### 2.5.2. Western Blot

Western blot was performed on tissue samples as described previously [36]. Briefly, aortas were homogenized by Tissue Lyser II (#85300 Qiagen, Germantown, MD, USA), sonicated, and centrifuged. Electrophoresis (50 μg of protein/sample) was carried out as described [36]. All experiments were performed at least three times.

#### 2.5.3. RNA Isolation and Quantitative RT-PCR

RNA isolation and quantitative RT-PCR (qRT-PCR) were performed on tissue samples as described previously [36]. After samples homogenization using the Tissue Lyser II (#85300 Qiagen), total RNA was purified using RNeasy Plus Kit (#74134 Qiagen) following manufacturer’s instructions. In total, 1 μg of RNA was reverse transcribed by Quanti Tect Reverse Transcription Kit (#205313 Qiagen), and quantitative RT-PCR was performed using Quanti Nova SYBR Green PCR Kit (#208056 Qiagen) in a Rotor-Gene qPCR machine (Qiagen). All experiments were performed at least three times.

#### 2.5.4. Tube Formation from Vessel Rings

Vessel sprouting form rat aorta was evaluated as described previously [36]. Rat aorta were cut in rings (1–2 mm) under sterile conditions. Vessels rings were included in a fibrin gel and after 24 h VEGF was added (25 ng/mL). The organ culture was kept at 37 °C, 5% CO_2_. Quantitative analysis was performed at day 3. Vessels were captured (Nikon Eclipse E400 and camera Nikon DS-5MC), images were converted in black and white to subtract the background of fibrin gel, and sprouting was manually measured by the means of an ocular grid as vessel length ± SD. Each experimental point was run in triplicate.

### 2.6. Statistical Analysis

Chemical data are expressed as the means of three independent experiments. The significance of differences among means was determined by one-way ANOVA (CoStat, Version 6.451, CoHort Software, Pacific Grove, CA, USA). Comparisons among means were performed by the Tukey’s test (*p* < 0.05).

Regarding in vivo evaluation of nutraceutical value of Phenol-Enriched ROOs, as well as the mitochondrial membrane potential, five different animals were used and data were expressed as mean ± SEM and one-way ANOVA followed by Bonferroni’s post hoc test used to compare groups for statistical differences (*p* < 0.05). All statistical procedures regarding in vivo evaluations were performed by commercial software (GraphPad Prism, version 5.0 from GraphPad Software Inc., San Diego, CA, USA).

Regarding Western blot and qPCR analysis, the results are the means ± SD of at least three independent experiments. The significance of differences among means was determined by one-way ANOVA with Tukey post-test analysis (GraphPad Prism, Version 5.0; *p* < 0.05).

## 3. Results and Discussion

### 3.1. Chemical Characterization of Phenol-Enriched ROOs (PE-ROOs)

#### 3.1.1. Quality Parameters

To verify the suitability of the proposed methods for the enrichment of ROO, the total phenols content as well as the values of the general quality parameters (free acidity, peroxide value, K_232_, and K_270_) of the 12 PE-ROOs were evaluated (Table 3) and compared with data determined for the control (ROO).

As reported in Table 3, the values of the quality parameters determined in all the PE-ROOs were within the range for the olive oil and makes them edible (FFA ≤ 1% oleic acid kg/kg; PV ≤ 15 meq O_2_/kg; K_270_ ≤ 0.90) [35]. According to ANOVA, among these general quality indexes K_232_ was the only parameter that showed some difference statistically significant (*p* ≤ 0.001) among treatments, with the highest value when the extraction was carried out using pomace and solvent B.

The phenolic content (Table 3B) significantly increased (*p* ≤ 0.001) in all prototypes PE-ROOs compared with the ROO used as reference and, for all the PE-ROOs, the values assumed appear close to those indicated for several high quality extra-virgin olive oils labeled with IGP appellation [41]. In the operating conditions adopted, according to ANOVA the best results were obtained when olive leaves were used as a source of phenols, regardless of the chemical composition of the solvent utilized for the extraction. As regards the solvent, the results showed significantly (*p* ≤ 0.001) different efficiency for the ROO fortification depending on the matrix used: for pomace, ethanol seems to be the best solvent according to the total phenols content; on the contrary, for grape marc and olive leaves the mix Ethanol/Hexane (1:1 kg/kg) was elected the best extracting solution, probably for a synergic effect between the two solvents [12]; always considering grape marc and olive leaves as sources for phenolic recovering, the mix ROO/Ethanol (1:1 kg/kg) also showed a good extracting power, representing a good compromise also considering its reduced toxicity.

In particular, when food grade solvent mixture was used (extract A and extract B), the concentration of total phenols in the enriched oils was 5.3-fold greater than that determined in the starting ROO when extract A (Ethanol 95.0%) was used; moreover, this value increased to reach 5.7 when extract B (ROO/Ethanol (1:1 kg/kg)) was used for the oil enrichment.

As shown in Table 3, the intensity of bitterness (IB) was directly proportional to the total phenolic content and the differences among values showed a significant level corresponding to a *p* value ≤ 0.001. Additionally, in contrast with the PE-OLs, pomace and grape marc samples could be classified as non-bitter or almost imperceptibly bitter oils, as they showed a value of IB ≤ 2.5, that was assumed as a reference point to indicate by chemical analysis the appearance of some bitter taste [39]. Further studies are needed to perform a proper panel test to confirm this evidence by sensory characterization as well.

According to ANOVA together with all the above discussed considerations, solution B (ROO/Ethanol (1:1 kg/kg)) was selected as the best solvent in terms of its safety, together with its efficacy for the increasing of phenolic content in ROO. For these reasons, all the PE-ROOs obtained by solution B (named PE-P-B, PE-GM-B, PE-OL-B) together with the ROO utilized as control were further characterized to individuate their UHPLC-HR-ESI-MS profiles and utilized to evaluate their nutraceutical properties in rats with high fat diet-induced-metabolic syndrome and oxidative stress.

#### 3.1.2. UHPLC-HR-ESI-MS Profiles

**Figure 2:**
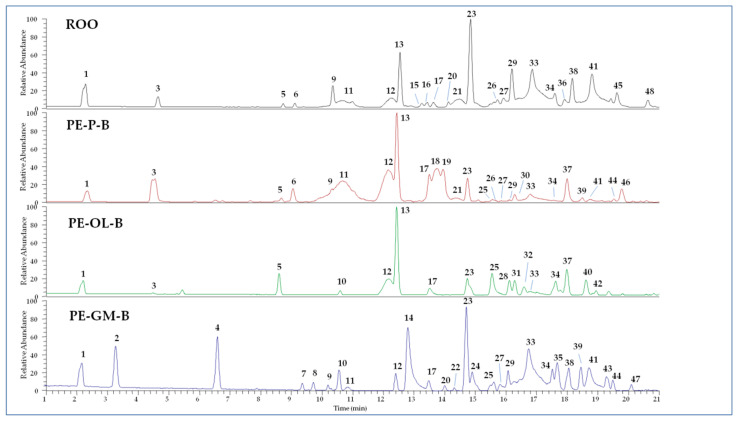
UHPLC-HR-ESI-MS profiles of extracts from refined olive oil (ROO) control, phenol-enriched ROO from olive pomace (PE-P-B), olive leaves (PE-OL-B), and grape marc (PE-GM-B). Peak data are listed in Table S1.

The polar fractions of PE-ROOs obtained by extraction with solvent B were subjected to qualitative analyses by UHPLC-HR-ESI-MS. The obtained chromatographic profiles operating in negative ion mode are showed in Figure 2, while detected compounds are described in Table S1. Compounds were tentatively identified according to MS and literature data [37,42,43,44,45,46,47,48]. Several substances were not completely identified but based on the MS fragmentation patterns their structure was correlated to some principal skeletons. Other peaks remained completely unidentified.

The ROO control is characterized by the presence of hydroxytyrosol and its derivatives, together with well-known secoiridoids, such as elenolic acid and both oleuropein and ligstroside aglycone derivatives, occurring as different isomers. Among flavonoids, luteolin (compound **17**) was detected. These results are in agreement with the chemical profile of olive oil reported by previous studies [36,48].

Compared to the ROO control, PE-P-B showed a very similar chemical composition, but also some modifications. In particular, two different forms of secoiridoids were detected, supposed to be ligstroside (compound **18**) and oleuropein derivative (compound **19**) on the basis of MS/MS data. In addition, compounds **37** and **46** were proposed as dihydrohybenzoic acid derivatives based on the observation of characteristic fragment ions at *m/z* 153.05, 109.06, 91.05. However, compounds **29**, **33**, **38**, **41**, and **45** (oleuropein aglycon derivatives) were revealed in PE-P-B in small amount compared to the control.

Similarly, the PE-OL-B extract lacked many secoiridoids observed in ROO control, such as elenolic acid (compound **11**) and related forms (compounds **3** and **9**), together with oleuropein aglycone derivatives (compounds **29**, **33**, **38**, **41**, and **45**). On the other hand, hydroxytyrosol (compound **1**) and its derivatives (compounds **5** and **13**) are well represented. The extract is also characterized by the presence of components that are absent in the control, such as secoiridoids **28** and **31**, the flavonoid methoxyluteolin (compound **32**), and two unidentified molecules (compounds **37** and **40**) whose structures seem to be correlated to dihydroxybenzoic acid derivatives. Olive leaves are known to be rich in secoiridoids (oleuropein and ligstroside derivatives) as well as in benzoic acid derivatives [49].

The chemical profile of PE-GM-B extract differed strongly from control one, preserving mainly hydroxytyrosol (compound **1**), hydroxytyrosol acetate (compound **12**), oleacin (compound **23**), and oleuropein aglycones (compounds **29**, **33**, **38**, and **41**). Many components were derived from grape marc, such as isopropyl malic acid (compound **2**), gallic acid ethyl ester (compound **4**), dihydroxybenzoic acid ethylester (compound **7**), quercetin (compound **8**), and its hexoside (compound **14**), azelaic acid (compound **10**), and kaempferol (compound **24**) [47].

### 3.2. In Vivo Evaluation of Nutraceutical Value of Phenol-Enriched ROOs (PE-ROOs)

#### 3.2.1. Effects of Supplementation with Olive Oils on Body Weight Gain in HFD-Fed Rats

**Figure 3:**
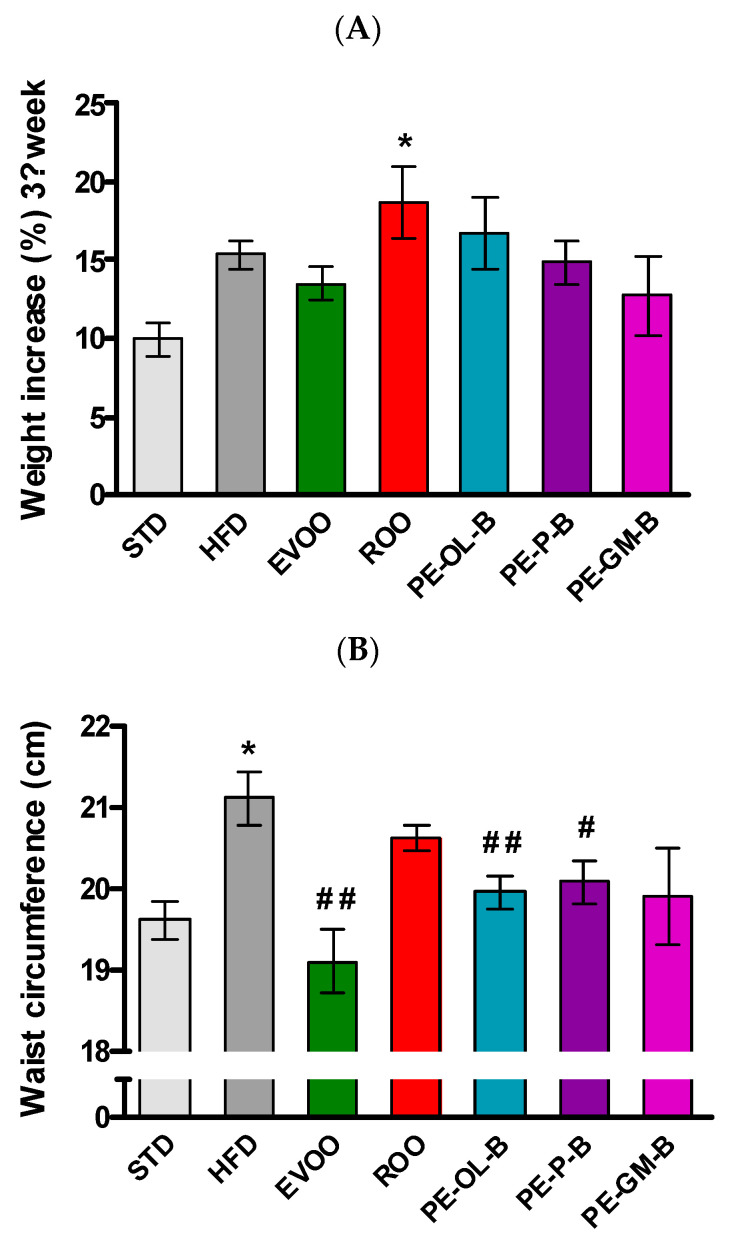
(**A**) Body weight gain of the animals at the third week of treatment (end of treatment). (**B**) waist circumference measured at the end of treatment. * indicates a statistically significant difference between the HFD group and the STD group. # indicates statistically significant difference vs. HFD. Single symbol corresponds to *p* ≤ 0.05. Double symbol corresponds to *p* ≤ 0.01.

In order to evaluate the nutraceutical value of the above PE-ROOs in vivo experiments were performed. In particular, a high fat diet is described as a well-known preclinical model of metabolic disorder. A diet with high levels of saturated fats (HFD) contributes to significantly (*p* ≤ 0.05) increase body weight. Indeed, a body weight percentage increase of 10.0% ± 1.0% occurs in animals fed with STD for 3 weeks, while animals fed with the HFD of 15.0% ± 0.9%. Conversely, animals supplemented with EVOO showed a slightly reduced (not statistically significant) body weight gain (13.0% ± 1.0%). As expected, at the third week, animals fed with HFD + ROO showed a marked (*p* ≤ 0.05) increase in body weight gain (18.0% ± 2.0%). Likewise, animals supplemented with novel products obtained by enrichment of ROO with by-products of olive oil and wine-making industry, overall showed a profile like ROO, and without relevant effects (Figure 3A).

In accordance with the body weight gain, at the third week a significant increase of waist circumference and BMI were reported in HFD-fed rats. EVOO supplementation, as previously reported [36], significantly (*p* ≤ 0.01) contributed to contain these parameters, while ROO supplementation was without significant effects. The novel products of olive oils (PE-OL-B, PE-GM-B, and PE-P-B) improved this parameter, but only the supplementation with PE-OL-B and PE-P-B more evidently and significantly (*p* ≤ 0.01 and *p* ≤ 0.05, respectively) contained the waist circumference (Figure 3B). Similar results were observed for the BMI parameter (data not shown).

**Figure 4:**
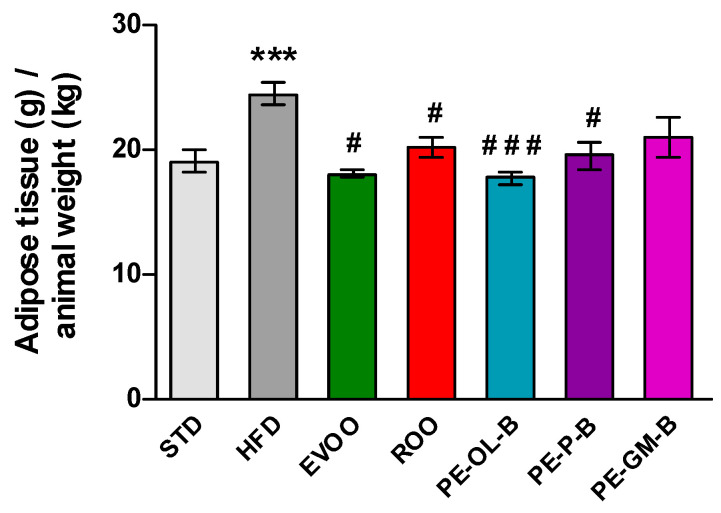
Ratio of adipose tissue (g) in relation to animal weight (kg) after 3 weeks of treatment with different diets. * indicates a statistically significant difference between the HFD group and the STD group. # indicates statistically significant difference vs. HFD. Single symbol corresponds to *p* ≤ 0.05. Triple symbol corresponds to *p* ≤ 0.005.

Beside the body weight, HFD promoted a significant (*p* ≤ 0.005) increase of visceral adipose tissue if compared with the STD group, while EVOO supplementation brought this parameter back to the levels of the STD group. Surprisingly, ROO supplementation also significantly contained adipose mass and the three novel ROO-transformed oils showed a similar behavior. Interestingly, PE-OL-B achieved a more evident and significant effect if compared with ROO (Figure 4).

**Figure 5:**
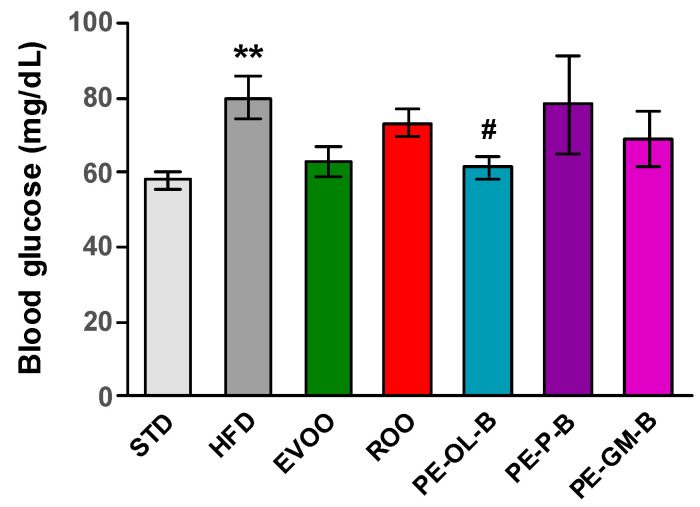
Fasting blood glucose levels (mg/dL) after 3 weeks of treatment with different diets. * indicates a statistically significant difference between the HFD group and the STD group. # indicates statistically significant difference vs. HFD. Single symbol corresponds to *p* ≤ 0.05. Double symbol corresponds to *p* ≤ 0.01.

After 3 weeks of treatment, fasting blood glucose was measured and significantly (*p* ≤ 0.01) higher levels of glycemia were reported; though it was indeed not possible to highlight a diabetic condition, an alteration of glucose homeostasis was evident (Figure 5). EVOO supplementation reduced the glucose levels after the treatment, while ROO only modestly made it. Notably, dietary intake of PE-OL-B significantly (*p* ≤ 0.05) reduced glucose levels coming back to the STD group levels. On the contrary PE-P-B and PE-GM-B did not positively influence this parameter (Figure 5).

**Figure 6:**
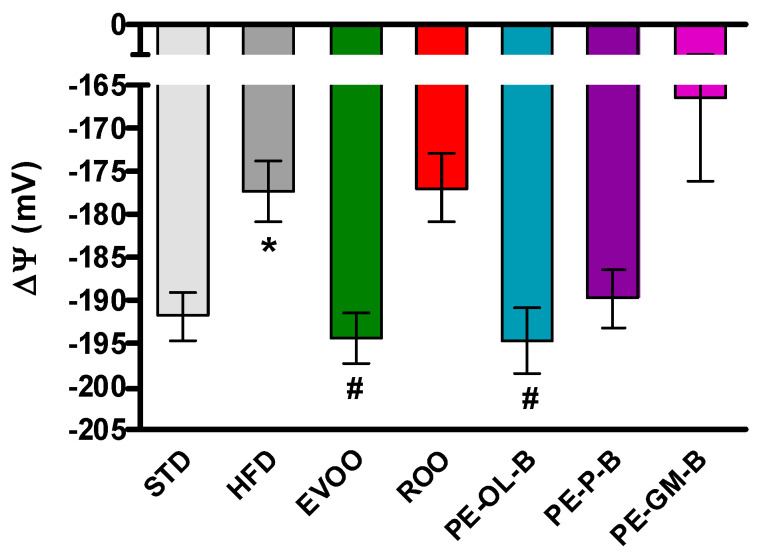
Changes in mitochondrial membrane potential in the different groups. * indicates a statistically significant difference between the HFD group and the STD group. # indicates statistically significant difference vs. HFD. Single symbol corresponds to *p* < 0.05.

Finally, the myocardial tissue was used to evaluate the mitochondrial membrane potential. The mitochondrial membrane potential is a parameter closely correlated with the metabolic efficiency of heart mitochondria and therefore with their ability to produce ATP and tolerate insults, as can be observed in animals with a condition like metabolic disorder. Under physiological conditions ΔΨ is about −190 mV, while after 3 weeks of a HFD diet ΔΨ appeared to be significantly depolarized (about −175 mV). The addition of EVOO reported the ΔΨ to the standard values, while ROO did not modify the HFD-induced impairment. Interestingly, the use of PE-OL-B for supplementation revealed an interesting significant (*p* ≤ 0.05) beneficial effect on mitochondrial function, showing a ΔΨ value superimposable to that of EVOO (Figure 6). These results suggested that PE-OL-B is the most promising product developed with this innovative technique.

#### 3.2.2. PE-ROOs on Aortic Vessel Inflammatory and Oxidative Stress Markers

**Figure 7:**
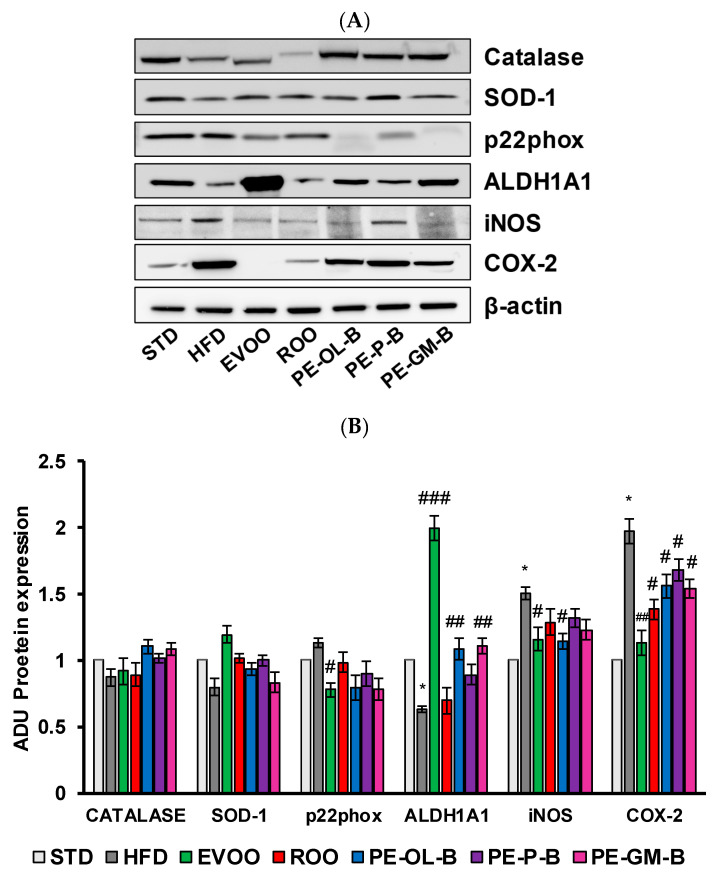
Diet supplementation of HFD fed rats with EVOO, ROO, PE-OL-B, PE-P-B, and PE-GM-B affects the expression of inflammatory and oxidative stress proteins in aortic vessel tissues. (**A**) Western blot analysis of oxidative stress factors (Catalase, SOD-1, p22phox, and ALDH1A1) and inflammatory factors (iNOS, COX-2) in the aorta tissues. (**B**) Quantification of Western blots. Data are determined by ratio of arbitrary density unit (ADU) target protein/β-actin, and reported as fold change vs. STD which was assigned to 1. Data are means ± SD (*n* = 3). * *p* ≤ 0.05 vs. STD; # *p* ≤ 0.05; ## *p* ≤ 0.01 vs. HFD; ### *p* ≤ 0.001 vs. HFD.

High dietary fat intake and high blood glucose are closely associated with cardiovascular risk factors, including the oxidative stress and impaired antioxidants defense mechanisms, and inflammation in micro- and macro-vessels [50,51]. Here, rat aortic tissues were used to measure the protein expression and mRNA levels of Catalase, superoxide dismutase-1 (SOD-1), p22phox, aldehyde dehydrogenase1A1 (ALDH1A1), inducible nitric oxide synthase (iNOS), and cycloxigenase-2 (COX-2) (Figure 7A,B) via Western blot and qPCR assays. In accordance with an increase of the body weight and glucose, HFD significantly (*p* ≤ 0.05) increased the expression of COX-2 (2 fold vs. STD) and iNOS proteins (1.5 fold vs. STD) in rat aortic tissues (Figure 7A,B). As previously reported, EVOO cosupplementation reverted the inflammatory profile of aortic tissues and increased the tissue expression of antioxidant ALDH1A1 enzyme [36]. ROO showed no significant effect on iNOS expression, nor was it able to significantly reverse the effect of HFD on ALDH1A1 expression. Of note is the effect of the ROO on COX-2 expression, which was significantly (*p* ≤ 0.05) reduced when compared to the effect of the HFD. Similarly, the novel oils (PE-OL-B, PE-P-B, PE-GM-B) had a significant (*p* ≤ 0.05) impact on COX-2, while iNOS protein expression was brought back to basal level only after PE-OL-B treatment.

Constituents from PE-OL-B and PE-GM-B supplemented into ROO showed also protective effects against oxidative stress markers, by restoring ALDH1A1 protein expression in aortic tissues and declining p22phox expression. SOD-1 and Catalase protein levels were not significantly affected by HFD nor by HFD cosupplemented with ROO or PE-OL-B, PE-P-B, PE-GM-B.

**Figure 8:**
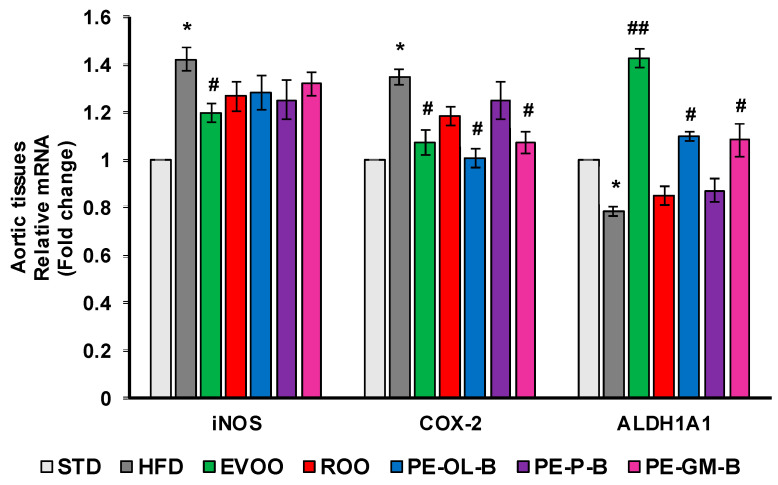
Diet supplementation of HFD fed rats with EVOO, ROO, PE-OL-B, PE-P-B, and PE-GM-B affects mRNA expression of inflammatory enzymes and ALDH1A1. Data are determined by the comparative Ct method (ΔΔCt) normalized to GAPDH expression and reported as fold change vs. STD group which was assigned to 1. Data are means ± SD (*n* = 3). * *p* ≤ 0.05 vs. STD; # *p* ≤ 0.05 vs. HFD; ## *p* ≤ 0.01 vs. HFD.

Consistently, at transcription level, we observed a significant (*p* ≤ 0.05) increase of iNOS and COX-2 expression in aortic tissues of rats fed with HFD, which were blunted by the cosupplement with EVOO (Figure 8). Similarly, PE-OL-B and PE-GM-B decreased COX-2 gene expression, while ROO had no effect. Furthermore, although we observed a trend toward a decrease, ROO and the novel olive oils did not significantly change iNOS expression at gene level (Figure 8).

HFD also significantly (*p* ≤ 0.05) reduced the transcription of ALDH1A1 gene in aortic tissues, and EVOO restored its expression at the levels of the STD group (Figure 8). Notably, dietary intake of PE-OL-B and PE-GM-B significantly (*p* ≤ 0.05) increased ALDH1A1 expression coming back to the EVOO group levels.

#### 3.2.3. PE-ROOs on Aortic Endothelial Function

**Figure 9:**
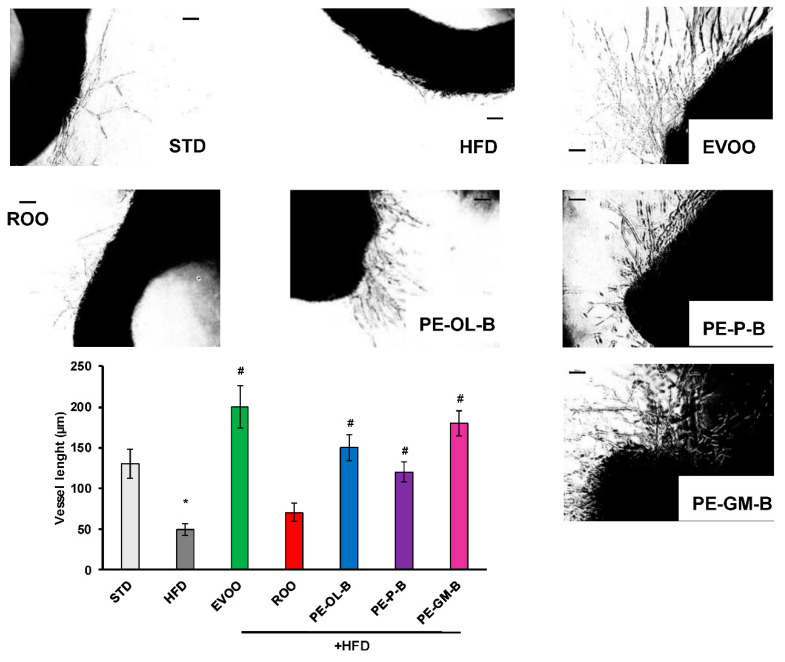
Diet supplementation of HFD fed rats with EVOO, ROO, PE-OL-B, PE-P-B, and PE-GM-B affects vessel sprouting formation from aortic rings. Aorta rings were treated with VEGF (25 ng/mL). Vessel sprouting formation was determined as the number of grid units covering the surface occupied by pseudo-capillaries in each well and expressed as vessel length. *n* = 4 rats. * *p* ≤ 0.05 vs. STD; # *p* ≤ 0.05 vs. HFD. Each experimental point is run at least in triplicate. Scale bar 100 µm.

To investigate the contribution of ROO, PE-OL-B, PE-P-B, PE-GM-B on the endothelial function, cells sprouting activity was performed. As shown in Figure 9, aortic rings of the STD rat group developed a net of microvessels in response to VEGF (25 ng/mL). HFD significantly (*p* ≤ 0.05) reduced the vessel sprouting formation which was reverted by EVOO supplementation. ROO had no significant effect, while PE-OL-B and PE-P-B displayed the ability to revert the negative effect of HFD on the outgrowths of branching microvessels. Surprisingly, PE-GM-B showed the greater induction of vessel sprouting. Taken together these data demonstrated a significant (*p* ≤ 0.05) protective effect of novel oils on endothelial functionality.

## 4. Conclusions

According to Reboredo-Rodriguez and co-workers [14] by 2035 70 million people will be aged >65 years old, thus the prevention of pathologies by diet is an important public health challenge in order to reduce the morbidity and mortality and the cost to society. Besides, the role played in health prevention by different types of olive oils appear deeply related with their content in minor compounds with antioxidant and anti-inflammatory properties [52].

In recent years refined olive oils are gaining importance in the food industry even thanks to the increasing importance of olive oil in the global market. As ROOs generally show reduced or absent minor components because of the refining process, some different strategies to produce functional ROOs with improved nutraceutical value were recently investigated [8,12,25,53]. When the content in antioxidant and anti-inflammatory compounds was significantly increased, the enriched ROOs generally showed an extended shelf life as well as an encouraging behavior in counteracting health degeneration in both in vivo and in vitro models.

In this context, in the present research, an innovative method to significantly improve the nutraceutical value of refined olive oil was developed and discussed. According to a circular economy concept the feasibility of the utilization of three different food by-products (namely olive leaves, pomace, and grape marc) as a source of natural phenolic compounds was verified and critically discussed and, to the best of our knowledges, no data are available in literature in this specific topic. Moreover, the utilization of a mixture ROO/Ethanol (1:1 kg/kg) as extraction solvent was also tested.

In conclusion all prototypes of phenol-enriched refined olive oils (PE-ROOs) showed a significantly improved phenolic content compared with the refined olive oil (ROO), used as reference together with a specific pattern of phenolic compounds as a function of the food waste utilized for the oil enrichment. Thanks to this innovative process, the enriched refined olive oils can reach a concentration of total phenols close to those indicated for several high-quality extra-virgin olive oils labeled with IGP appellation.

In the operating conditions adopted, the best results were obtained when olive leaves were used as source of phenols, regardless of the chemical composition of the solvent utilized for the extraction. Furthermore, while hexane was confirmed as a good solvent for the extraction of phenols compounds soluble in oil, the mix ROO/Ethanol (1:1 kg/kg) also showed a good extracting power from olive leaves together with a reduced toxicity and lower environmental impact. Beyond the technological approach, interestingly this work demonstrated that the novel refined enriched olive oils (PE-OL-B, PE-GM-B, and PE-P-B) showed a greater nutraceutical value compared to ROO, utilized as control. In fact, they reduced the impact of a high fat diet-induced-metabolic disorder and oxidative stress in rats. Among all, PE-OL-B, closely followed by PE-GM-B, significantly reduced the increase in adipose mass, waist circumference, BMI parameters, glucose level, and improved the mitochondrial membrane potential. Consistently, in aortic vessels, the two PE-ROOs reduced the expression of inflammatory iNOS and COX-2 enzymes and increased that of the antioxidant ALDH1A1 enzyme. Furthermore, the use of PE-OL-B for supplementation revealed an interesting beneficial effect on mitochondrial function and a significant protective effect on endothelial functionality.

Starting from this evidence, in future, perspective panel tests followed by a consumer test should be assessed to define the sensorial profile of the selected novel enriched oil (PE-OL-B) as well as the consumer acceptability both at national and international level. A set of experimental runs to determine the shelf life of the PE-OL-B and to individuate the best operating conditions to be adopted for storage should also be performed. Finally, the scaling up to produce the proposed novel oil at prototypal level first and industrial level after should be set up, together with a specific analysis of the costs of the innovative process.

## Figures and Tables

**Table 1:** Total phenols of pomace, grape marc, and olive leaves. Data are expressed as mean ± confidence interval (*n* = 3) at *p* ≤ 0.05.

Parameter	Pomace	Grape Marc	Olive Leaves
Total phenols (g gallic acid/L)	(3.5 ± 0.1)	(1.5 ± 0.2)	(3.1 ± 0.2)

**Table 2:** Sample codes.

Sample	Description
ROO	Refined Olive Oil (Control)
PE-P-A	ROO + phenol extracted from Pomace by sol. A
PE-P-B	ROO + phenol extracted from Pomace by sol. B
PE-P-C	ROO + phenol extracted from Pomace by sol. C
PE-P-D	ROO + phenol extracted from Pomace by sol. D
PE-GM-A	ROO + phenol extracted from Grape Marc by sol. A
PE-GM-B	ROO + phenol extracted from Grape Marc by sol. B
PE-GM-C	ROO + phenol extracted from Grape Marc by sol. C
PE-GM-D	ROO + phenol extracted from Grape Marc by sol. D
PE-OL-A	ROO + phenol extracted from Olive Leaves by sol. A
PE-OL-B	ROO + phenol extracted from Olive Leaves by sol. B
PE-OL-C	ROO + phenol extracted from Olive Leaves by sol. C
PE-OL-D	ROO + phenol extracted from Olive Leaves by sol. D

**Table 3:** (**A**) Chemical characterization (free fatty acids (FFA), peroxide value (PV), and spectrophotometric indexes (K_270_ and K_232_)) of phenol-enriched ROOs; (**B**) phenolic content, antioxidant capacity, and intensity of bitterness of phenol-enriched ROOs. Each value represents mean ± standard deviation (*n* = 3).

(**A**)					
**Sample**		**FFA (% Oleic Acid kg/kg)** **n.s.^1^**	**PV (mEq.O_2_/kg Oil)** **n.s.**	K_232_ ***	**K_270_** **n.s.**
Control	Solvent ^2^	(0.18 ± 0.01)	(7.62 ± 0.05)	(1.60 ± 0.01) ^b^	(0.62 ± 0.01)
Pomace	A	(0.18 ± 0.01)	(8.02 ± 0.01)	(1.60 ± 0.01) ^b^	(0.62 ± 0.01)
	B	(0.18 ± 0.01)	(8.01 ± 0.01)	(1.73 ± 0.01) ^a^	(0.63 ± 0.01)
	C	(0.18 ± 0.01)	(8.00 ± 0.01)	(1.66 ± 0.01) ^ab^	(0.68 ± 0.03)
	D	(0.18 ± 0.01)	(8.03 ± 0.02)	(1.68 ± 0.02) ^ab^	(0.670.01)
Grape Marc	A	(0.18 ± 0.01)	(8.01 ± 0.01)	(1.66 ± 0.01) ^ab^	(0.68 ± 0.02)
	B	(0.18 ± 0.01)	(8.00 ± 0.01)	(1.67 ± 0.01) ^ab^	(0.63 ± 0.01)
	C	(0.18 ± 0.01)	(8.01 ± 0.01)	(1.68 ± 0.01) ^ab^	(0.62 ± 0.01)
	D	(0.18 ± 0.01)	(8.02 ± 0.02)	(1.68 ± 0.02) ^ab^	(0.63 ± 0.01)
Olive Leaves	A	(0.18 ± 0.01)	(8.02 ± 0.01)	(1.65 ± 0.01) ^ab^	(0.67 ± 0.02)
	B	(0.18 ± 0.01)	(8.03 ± 0.01)	(1.66 ± 0.01) ^ab^	(0.65 ± 0.01)
	C	(0.18 ± 0.01)	(8.01 ± 0.01)	(1.68 ± 0.01) ^bc^	(0.68 ± 0.03)
	D	(0.18 ± 0.01)	(8.00 ± 0.01)	(1.68 ± 0.02) ^bc^	(0.67 ± 0.02)
(**B**)					
**Sample**		Total Phenols (mg/kg Gallic Acid) *** ^1^	FRSC ABTS (μmol TEAC/mL) ***	FRSC DPPH (μmol TEAC/mL) ***	Intensity of Bitterness (IB) ***
Control	Solvent ^2^	(50 ± 1) ^g^	(0.38 ± 0.02) ^e^	(0.26 ± 0.01) ^e^	(0.00 ± 0.01) ^d^
Pomace	A	(210 ± 3) ^c^	(0.52 ± 0.01) ^d^	(0.41 ± 0.01) ^d^	(0.82 ± 0.01) ^cd^
	B	(260 ± 2) ^b^	(0.61 ± 0.02) ^c^	(0.50 ± 0.01) ^c^	(1.66 ± 0.02) ^bc^
	C	(143 ± 1) ^de^	(0.79 ± 0.03) ^b^	(0.65 ± 0.02) ^b^	(2.22 ± 0.01) ^b^
	D	(120 ± 1) ^e^	(0.41 ± 0.01) ^e^	(0.30 ± 0.01) ^e^	(1.70 ± 0.03) ^bc^
Grape Marc	A	(143 ± 1) ^de^	(0.38 ± 0.01) ^e^	(0.27 ± 0.01) ^e^	(0.50 ± 0.01) ^d^
	B	(90 ± 2) ^f^	(0.50 ± 0.02) ^d^	(0.42 ± 0.02) ^d^	(0.10 ± 0.01) ^d^
	C	(170 ± 1) ^d^	(0.38 ± 0.01) ^e^	(0.26 ± 0.01) ^e^	(0.18 ± 0.01) ^d^
	D	(75 ± 1) ^fg^	(0.38 ± 0.01) ^e^	(0.26 ± 0.01) ^e^	(0.17 ± 0.01) ^d^
Olive Leaves	A	(263 ± 2) ^b^	(0.62 ± 0.02) ^c^	(0.50 ± 0.01)	(4.75 ± 0.02) ^a^
	B	(287 ± 4) ^b^	(0.75 ± 0.02) ^b^	(0.62 ± 0.02) ^b^	(4.15 ± 0.01) ^a^
	C	(698 ± 5) ^a^	(0.97 ± 0.04) ^a^	(0.80 ± 0.02) ^a^	(4.45 ± 0.02) ^a^
	D	(220 ± 1) ^c^	(0.50 ± 0.01) ^d^	(0.40 ± 0.01) ^d^	(4.50 ± 0.01) ^a^

^1^ Significance level—***: *p* ≤ 0.001 (F = 15,521); ns: not significant (*p* > 0.05). Within the same column, parameters sharing the different letters have a significantly different mean value.^2^ Composition of different solvent solutions: Ethanol (95.0%), named sol. A; ROO/Ethanol (1:1 kg/kg), named sol. B; Ethanol/Hexane (1:1 kg/kg), named sol. C, Hexane (97.0%) named sol. D.

## Supplementary Materials

The following are available online at https://www.mdpi.com/2304-8158/9/10/1390/s1, Table S1: UHPLC-HR-ESI-MS/MS data of components.

## Abbreviations

3:4-DHPEA	3,4-dihydroxyphenyl ethanol,
ABTS	2,2′-azino-bis(3-ethylbenzothiazoline-6-sulfonic acid)
ADU	arbitrary density unit
ALDH1A1	aldehyde dehydrogenase 1A1
BMI	Body mass index
COX-2	cycloxigenase-2
DAFE	Department of Agriculture, Food and Environment
DPPH	2,2-diphenyl-1-picrylhydrazyl
EGTA	ethylene glycol-bis(β-aminoethyl ether)-N,N,N′,N′-tetraacetic acid
ESI	electrospray ionization
EVOO	extra-virgin olive oil
FFA	free fatty acids
FRSC	free-radical scavenging capacity
GAPDH	glyceraldehyde-3-phosphate dehydrogenase
GM	grape marc
HCD	collisionally activated dissociation
HFD	high fat diet
HR-MS	high resolution-mass spectrometry
IB	intensity of bitterness
iNOS	inducible nitric oxide synthase
MedDiet	Mediterranean diet
MetS	metabolic syndrome
MUFA	monounsaturated fatty acids
OL	olive leaves
OO	olive oil
P	pomace
PE	phenol-enriched
PGI	protected geographical indication
PUFA	polyunsaturated fatty acids
PV	peroxide value
qRT-PCR	quantitative real time polymerase chain reaction
ROO	refined olive oil
SD	standard deviation
SEM	standard error of the mean
SOD-1	superoxide dismutase-1
STD	standard diet
TEAC	Trolox equivalent antioxidant capacity
TPP^+^	cation tetraphenylphosphonium
*t*_R_	retention time
UHPLC	ultra-high-performance liquid chromatography
VEGF	vascular endothelial growth factor
VOO	virgin olive oil
ΔΔCt	comparative Ct method
