# Supplementary material for: By-Products from Winemaking and Olive Mill Value Chains for the Enrichment of Refined Olive Oil: Technological Challenges and Nutraceutical Features
Source: Foods. 2020 Oct 1;9(10):1390. doi: 10.3390/foods9101390 (PMC7601883; doi:10.3390/foods9101390)
Supplement: Supplementary file 1 [file foods-09-01390-s001.pdf]

Table S1. UHPLC-HR-ESI-MS/MS data of components **1–48** detected in refined olive oil (ROO) control (sample a), phenol-enriched ROO from pomace (PE-P-B, sample b), olive leaves (PE-OL-B, sample c), and grape marc (PE-GM-B, sample d).

Peak [1]	Compound [2]	t_R (min)	HR-[M–H]⁻ (*m*/*z*)	HR-MS/MS Product Ions (*m*/*z*)	Error (ppm)	Sample
	Secoiridoids					
**1**	Hydroxytyrosol (3,4-DHPEA)	2.2	153.0550	123.04	−4.57	a–d
**3**	Hydroxylated decarboxymethyl elenolic acid	4.6	199.0606	155.07, 111.08, 95.05, 85.03, 69.03, 59.01	−3.01	a–c
**5**	Hydroxytyrosol acetate, isomer I	8.7	195.0657	59.01	−3.08	a–c
**6**	Hydroxylated elenolic acid, isomer I	9.1	257.0665	181.05, 137.06, 109.07, 95.05	−0.77	a,b
**9**	Hydroxylated elenolic acid, isomer II	10.3	257.0665	181.05, 137.06, 109.07, 95.05	−0.77	a,b,d
**11**	Elenolic acid	10.8	241.0715	139.00, 127.04, 101.02, 95.05, 69.00	−3.32	a,b,d
**12**	Hydroxytyrosol acetate	12.3	195.0657	59.01	−3.08	a–c
**13**	10-Hydroxy-oleuropein aglycone decarboxymethyl	12.6	335.1130	199.06, 155.07, 111.08, 59.01	−1.79	a–d
**15**	Oleuropein aglycone, isomer I	13.3	377.1236	307.08, 275.06, 139.04, 111.01, 95.04	−1.59	a
**16**	Oleuropein aglycone, isomer II	13.4	377.1236	307.08, 275.06, 139.04, 111.01, 95.04	−1.59	a
**21**	Lygstroside aglycone decarboxymethyl (oleocanthal)	14.6	303.1232	165.06, 137.06, 69.03, 59.01	−1.98	a,b
**23**	Oleacein, isomer I (oleuropein aglycone decarboxymethyl)	14.9	319.1182	199.06, 181.05, 155.07, 111.01, 85.03, 59.01	−1.57	a–d
**26**	Ligstroside aglycone, isomer I	15.7	361.1288	291.09, 259.10, 101.02, 69.00, 59.01	−1.38	a,b
**27**	Oleuropein aglycone, isomer II	15.9	377.1236	307.08, 275.06, 139.04, 111.01, 95.04	−1.59	a,b,d
**29**	Hydroxy oleuropein aglycone, isomer I	16.2	393.1186	317.02, 181.05, 153.06, 137.06, 109.06	−1.27	a,b,d
**33**	Oleuropein aglycone, isomer III	17.0	377.1236	307.08, 275.06, 139.04, 111.01, 95.05	−1.59	a–d
**36**	Ligstroside aglycone, isomer II	17.9	361.1288	291.09, 259.10, 101.02, 69.00	−1.38	a
**38**	Oleuropein aglycone, isomer IV	18.2	377.1234	333.13, 301.11, 181.05, 137.06, 109.07, 59.01	−2.12	a,d
**39**	Dehydro oleuropein aglycone	18.5	375.1082	239.05, 195.07, 179.03, 137.02	−0−80	b,d
**41**	Oleuropein aglycone, isomer V	18.8	361.1287	291.09, 259.10, 101.02, 69.00	−1.66	a,b
**45**	Oleuropein aglycon derivative	19.6	349.1287	213.08, 181.05, 137.06, 109.06	−1.72	a
	Flavonoids					
8	Quercetin 3-*O*-glucoside/galactoside	9.8	463.0881	300.03, 271.02, 255.03, 151.00	−0.22	d

**14**	Quercetin	12.8	301.0349	273.04, 179.00, 151.00, 121.03	−1.66	d
**17**	Luteolin	13.6	285.0400	199.04, 151.00, 133.03	−1.75	a–d
**24**	Kaempferol	14.9	285.0403	257.04, 229.05	−070	d
**25**	Apigenin	15.6	269.0452	225.05, 151.00, 117.03	−1.11	a–d
**32**	Methoxyluteolin	16.6	299.0557	284.03, 256.04, 155.04	−1.34	c
	Organic acids					
**2**	2-Isopropyl malic acid/2-isopropyl malic acid	3.3	175.0605	131.07, 115.04, 113.06, 85.07	−3.99	d
**4**	Gallic acid ethyl ester	6.6	197.0449	169.01, 125.02, 111.01	−3.04	d
**7**	Dihydroxybenzoic acid ethylester	9.4	181.0499	153.02, 109.03	−3.87	d
**10**	Azelaic acid	10.6	187.0969	169.09, 125.10, 97.06	−3.74	c,d
**42**	Dihydroxyhexadecanoic acid	18.9	287.2225	269.21, 241.22		c
	Unidentified compounds					
**18**	Ligstroside derivative	13.8	363.1447	139.08, 95.05, 69.03, 59.01		b
**19**	Oleuropein derivative	13.9	379.1395	199.06, 181.05, 151.04, 111.08, 95.05, 59.01		b
**20**	Hydroxytyrosol derivative, isomer I	14.1	365.1237	229.07, 185.08, 153.05, 109.06		a,d
**22**	Unidentified	14.4	169.0864	125.10, 83.05, 71.05, 55.02		d
**28**	Ligstroside aglycone derivative	16.0	439.1289	213.07, 181.05, 153.05, 137.06, 111.01, 95.05		c
**30**	Hydroxytyrosol derivative	16.3	291.1236	155.07, 111.08, 69.03, 59.01		b
**31**	Hydroxytyrosol derivative, isomer II	16.4	365.1600	229.11, 185.12, 153.09, 139.07, 121.06		c
**34**	Unidentified	17.6	293.1754	236.10, 221.15, 148.05, 71.01		a–d
**35**	Unidentified	17.7	329.2330	229.14, 211.13, 171.10, 139.11		d
**37**	Dihydroxybenzoic acid derivative	18.0	325.0845	153.05, 109.06, 91.05		b,c
**40**	Dihydroxybenzoic acid derivative	18.7	455.1706	385.13, 153.05, 109.06, 95.05		c
**43**	Unidentified	19.3	227.1648	209.15, 158.98, 130.98		d
**44**	Hydroxy oleuropein aglycone, isomer II	19.5	393.1917	257.14, 139.08, 109.06, 95.5, 59.01		b,d
**46**	Dihydroxybenzoic acid derivative	19.8	369.1105	333.13, 153.05, 109.06, 91.05		b
**47**	Unidentified	20.1	297.1703	279.16, 253.18, 235.17		d
**48**	Unidentified	20.7	387.2859	283.27, 102.05, 75.01		a

[1] Compound numbers correspond with peak numbers showed in Figure 2. [2] Tentatively identified based on MS/MS and literature data.
